# Emotional dysregulation in children with attention-deficit/hyperactivity disorder

**DOI:** 10.1007/s12402-016-0199-0

**Published:** 2016-06-14

**Authors:** Judy van Stralen

**Affiliations:** Center for Pediatric Excellence, Ottawa, ON Canada

**Keywords:** ADHD, Emotional dysregulation, Emotional lability, Deficient emotional self-regulation

## Abstract

Emotional dysregulation is increasingly recognized as a core feature of attention-deficit/hyperactivity disorder (ADHD). The purpose of the present systematic literature review was to identify published data related to the neuropsychology of emotional dysregulation in children with ADHD. The literature obtained is discussed in the contexts of deficits in emotional control, impairments in executive function, the emotional components of comorbidities, neurophysiological and autonomic correlates of emotional dysregulation, and the significance of multiple neuropsychological pathways of ADHD on emotional dysregulation. These various lines of evidence are used to create a patient-oriented conceptual model framework of the pathway from stimulus to inappropriate internalized (sadness, moodiness) or externalized (anger, aggressiveness) emotional responses. The article concludes by calling for continued research into the development of reliable and universally accepted measures of emotional dysregulation in order to provide children affected with ADHD, and their caregivers, some explanation for their emotional lability and, ultimately, to be used as tools to evaluate potential treatments.

## Introduction

Barry is a 10-year-old boy with attention-deficit/hyperactivity disorder (ADHD) who has been suspended from school. His father stays at home with him, but insists that Barry does a number of homework exercises. Barry understands the necessity and is doing the work, but becomes increasingly frustrated by the amount of work. Barry thinks he is doing the last exercise when his father indicates there is one more page of math to do. Barry’s heart starts to beat a little faster and he feels agitated. Just then the telephone rings; it is the school principal wishing to speak to Barry’s father about the suspension and reintegration process. Barry can hear only a little of the conversation but, already agitated, he becomes increasingly angry. His blood pressure rises, his face turns red, and he begins to throw things, first around his bedroom and then around the house. He yells and screams, disrupting the telephone call, and strikes out at his father in desperation. Finally, he retreats to his bedroom, destroying things along the route.

Equally, consider Rachel, a 7-year-old girl with the inattentive presentation of ADHD. Rachel is frustrated with not understanding some homework on which she is working. She feels overwhelmed by a project that is due in a few days. Rachel has kept her emotions inside, and her mother is not aware of her frustrations. Her mother asks her to clear the dinner table. Rachel does not respond at first, but when her mother asks her again, Rachel bursts into tears stating that her mother is always demanding so much of her.

ADHD is a neurodevelopmental disorder characterized by impulsivity, hyperactivity, and/or inattention according to the *Diagnostic and Statistical Manual of Mental Disorders 5th edition (DSM*-*5)* (American Psychiatric Association 2013). It has been recognized for many years, however, that many children with ADHD exhibit low frustration tolerance and explosive behavior (Laufer et al. 1957) and, increasingly, there are calls to re-assess the characteristics of emotional impulsiveness and deficient emotional self-regulation as core features, rather than simply associated aspects of the disorder (Barkley 1997a, 2010). Indeed, the childhood manifestations of emotional dysregulation are important criteria of the Wender Utah Rating Scale used in the diagnosis of adult ADHD (Ward et al. 1993). Initial efforts to develop a unified model of ADHD proposed that impaired behavioral inhibition leads to deficits in executive neuropsychological functions that depend on such inhibition, including working memory, self-regulation of affect–motivation–arousal, internalization of speech, and reconstitution (behavioral analysis and synthesis) (Barkley 1997b), although others have argued that impaired behavioral inhibition and executive function are independent of each other (Rhodes et al. 2005). Based on a discovery-based community algorithm, Karalunas et al. (2014) proposed three subtypes of ADHD: mild (normative emotional regulation); surgent (extreme levels of positive-approach motivation); and irritable (extreme levels of negative emotionality, anger, and poor soothability) (Karalunas et al. 2014). The three subtypes were independent of existing clinical groupings, showed stability over time, and were distinguished by unique patterns of cardiac physiological response, resting-state functional brain connectivity, and clinical outcomes.

According to Barkley’s model (Barkley 1997a, b), the deficits in inhibition when individuals with ADHD are faced with emotionally charged situations lead to greater emotional reactivity or emotional dysregulation, compared with those without ADHD. Dysregulated emotion is characterized by excessive and rapidly shifting emotions, often associated with irritable and aggressive behavior (Stringaris 2011), and high rates of comorbid oppositional defiant disorder (Stringaris and Goodman 2009). For the purposes of the present review, we define emotional dysregulation as *an inability to modulate emotional responses, resulting in extreme responses of an internalizing or externalizing nature that would be considered inappropriate for the developmental age of the person*. In a recent review, the authors concluded that emotional dysregulation is highly prevalent in ADHD and is a major contributor to impairment, is associated with deficits in the recognition and/or allocation of attention to emotional stimuli, implicating deficits in the striato-amygdalo-medial prefrontal cortical network, and may be ameliorated by ADHD treatments (Shaw et al. 2014).

The purpose of the present article is to undertake a systematic literature review of clinical data relating to emotional dysregulation in children with ADHD, focusing on deficits in emotional inhibition and emotional self-regulation, and evidence of impaired executive function. To conclude, the article proposes a conceptual model of the construct of emotional dysregulation in ADHD and describes the importance of recognizing that emotional dysregulation is a common feature in children with ADHD.

## Literature search

**Fig. 1:**
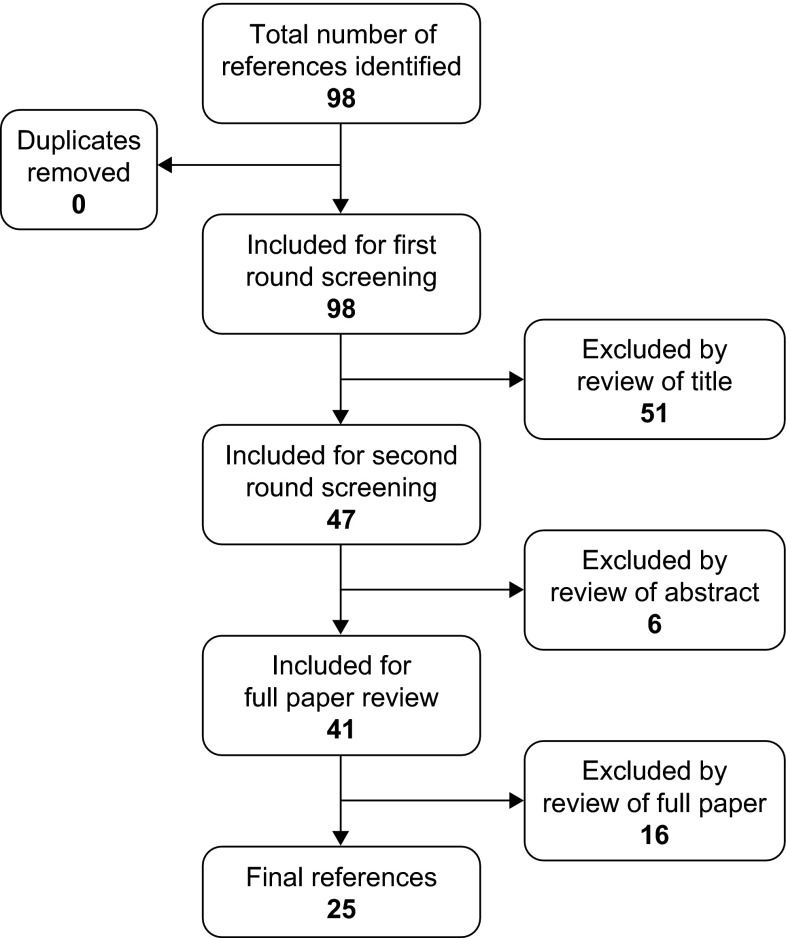
PRISMA (Preferred Reporting Items for Systematic Reviews and Meta-Analyses) flow diagram.

A PubMed search was performed on September 4, 2015, using the following terms: ((((((addh[MeSH Terms]) AND ((emotion OR emotional)) AND (regulation OR dysregulation))) AND Humans[Mesh] AND English[lang])) NOT review[Publication Type]) NOT adult. Articles were required to contain data related to the neuropsychology of emotional dysregulation in children with ADHD. Reviews, case studies, and commentaries were excluded, as were articles that were not specifically related to ADHD and emotional dysregulation (including articles describing studies in individuals in which ADHD was not the primary diagnosis). The search strategy is described in the Preferred Reporting Items for Systematic Reviews and Meta-Analyses (PRISMA) flow diagram (Fig. 1). The literature search identified 25 original articles (Table 1).

**Table 1:** Summary of search results

References	Study population	Measures	Conclusions
Anastopoulos et al. (2011)	Age 5–12 years *n* = 358 ADHD 74 % (ADHD-C, 52.8 %; ADHD-PI, 36.6 %; ADHD-HI, 10.6 %)	Parent-completed Conners’ Rating Scales–Revised Behavior assessment system for children–2nd edition	46.92 % of the children affected by ADHD displayed significantly elevated levels of emotional lability versus 15.38 % of those without this disorder Emotional lability was strongly associated with both aggression and depression; deficits in the self-regulation of emotion may serve as a marker for these comorbid outcomes
Beauchaine et al. (2013)	Age 4–6 years *n* = 99 (ADHD)	*Psychopathology measures* Child Symptom Inventory Child behavior checklist Eyberg Child Behavior Inventory Conners’ Parent Rating Scale–Revised Social Competence Scale *Cardiovascular measures* Cardiac pre-ejection period Respiratory sinus arrhythmia	Lengthened cardiac pre-ejection period at rest and reduced pre-ejection period reactivity to incentives led to higher scores on measures of conduct problems and aggression Lower baseline respiratory sinus arrhythmia and greater respiratory sinus arrhythmia withdrawal led to lower scores on prosocial behavior Greater respiratory sinus arrhythmia withdrawal led to lower scores on emotion regulation
Berlin et al. (2004)	Age 7–10 years Total, *n* = 63 ADHD, *n* = 21; ODD, *n* = 7; TS, *n* = 4; DCD, *n* = 3; dyslexia, *n* = 8; control, *n* = 42	Go/No-Go task Stroop-like task Kaufman hand movements test Puzzle cheating task Emotion regulation using a parental rating scale (Rydell et al. 2003) Story reconstruction test	Children with ADHD differed significantly from controls with regard to measures of inhibition as well as all other executive function measures, except repetition of hand movements In logistic regression models, three different measures (tapping inhibition, working memory, and emotion regulation) were shown to be significant independent predictors of group membership Children with ADHD differed from controls when studying mean group differences in all of the components in Barkley’s model; the measures discriminated well between groups; the results concerning independent effects pointed to inhibition and working memory as salient aspects
Braaten and Rosen (2000)	Age 6–12 years ADHD, *n* = 24; controls, *n* = 19	Developmental Questionnaire (Gordon 1995) Peabody picture vocabulary test-revised Empathy response task (Ricard and Kamberk-Kilicci 1995) Emotions behavior checklist Emotional Intensity Scale for Children Emotional reactions to external Contingencies Scale for children	Children with ADHD were less likely than those without ADHD to match their emotion with the emotion of a child in a story eliciting negative emotions and gave significantly fewer character-centered interpretations in their descriptions of the character’s emotion Children with ADHD appear to show more negative emotion, particularly depression, anger, and guilt, than do children without ADHD; children with ADHD may be able to self-regulate their positive emotions but not negative emotions
Brotman et al. (2010)	Age 8–17 years ADHD, *n* = 18; BD, *n* = 43; SMD, *n* = 29; controls, *n* = 37	Children’s Depression Rating Scale Young Mania Rating Scale Whole brain blood-oxygen level-dependent functional MRI	Relative to healthy comparison participants, patients with ADHD manifested hyperactivation in the left, but not right, amygdala Significant group effect in the left amygdala, with patients with severe mood dysregulation showing hyperactivation While rating subjective fear of neutral faces, youths with ADHD demonstrated left amygdala hyperactivity relative to the other three groups, whereas youths with severe mood dysregulation demonstrated hypoactivity
Gow et al. (2013)	Age (StDev) ADHD, 14.46 (1.12) years Controls, 14.00 (1.10) years ADHD, *n* = 31; controls, *n* = 39	Event-related potentials Electroencephalograms Blood analysis	Children with ADHD had lower mean omega-3/6 levels and event-related potential abnormalities in emotion processing, independent of emotional valence relative to control children Lower omega-3 fatty acid levels were associated with impaired emotion processing in children with ADHD
Harty et al. (2009)	Age 7–11 years at initial diagnosis. ~10 years follow-up ADHD, *n* = 85 (ODD 52 %, CD 26 %); control, *n* = 83	Buss–Perry Aggression Questionnaire State-Trait Anger Expression Inventory-2	ADHD plus conduct disorder in childhood led to elevated levels of physical aggression when compared with controls and the ADHD-only group. ADHD plus oppositional defiant disorder led to elevated levels of verbal aggression compared with controls Adolescents diagnosed with ADHD and comorbid disruptive behavior disorders during childhood reported high levels of aggression associated with increased emotionality in the form of anger, but not hostile cognitions
Hulvershorn et al. (2014)	Age (StDev), 9.2 (2.0) years *n* = 63	Kiddie SADS–present and lifetime version Conners’ Teacher Rating Scale–revised, long version Conners’ Parent Rating Scale–revised, long version Resting-state functional MRI	Children with ADHD who were impaired by high emotional lability exhibited aberrant amygdala-cortical intrinsic functional connectivity, i.e., in regions associated with emotion regulation Findings suggested that a subset of youth with ADHD have specific disruptions in amygdala networks that underlie emotion regulation impairments Resting-state functional connectivity appears to be suitable for detecting emotion relevant differences in intrinsic functional connectivity in youth with ADHD
Jensen and Rosen (2004)	Age 6–15 years ADHD, *n* = 30; controls, *n* = 37 Depression, BD, OCD, FAS, etc. excluded	Disruptive Behavior Rating Scale–Parent Emotional intensity scale–parent version (Intensity Scale) Emotional reactions to external contingencies scale–parent version	Children with ADHD were rated as significantly more emotionally reactive to both immediate and future events than were children without ADHD Differences at both the immediate and future time periods were stronger in response to negative as opposed to positive emotional events In response to the consequences of their behavior, children with ADHD were rated as less emotionally reactive than children without ADHD
Maedgen and Carlson (2000)	Age 8–11 years Total ADHD, *n* = 30 (ADHD-C, *n* = 16; ADHD-I, *n* = 14); controls, *n* = 17	Children’s Assertive Behavior Scale Revised behavior problem checklist Duncan’s socioeconomic index of occupational status Wide range achievement test–third edition Wechsler Intelligence Scale for children–3rd edition Facial and non-facial coding	Children with ADHD–C were rated as showing more aggressive behavior; they displayed emotional dysregulation characterized by high intensity and high levels of both positive and negative behavior Children with ADHD–I displayed social passivity and showed deficits in social knowledge on the self-report measure but did not evidence problems in emotional regulation ADHD subtypes may benefit from different treatment approaches
Martel and Nigg (2006)	Age 6–12 years ADHD, *n* = 92 (ADHD-I, *n* = 24; ADHD-C, *n* = 68); borderline, *n* = 35; controls, *n* = 52	California child Q-sort Early Adolescent Temperament Questionnaire	Reactive Control was related to hyperactivity–impulsivity as rated by both parents and teachers. Negative Emotionality was related to oppositional defiance. Resiliency was primarily related to inattention–disorganization as rated by both parents and teachers; Effortful Control was related uniquely to inattention in parent but not teacher data Low levels of Reactive Control may have led to high levels of hyperactivity–impulsivity, and low levels of resiliency or Effortful Control may have been related to high levels of inattention Children with ADHD may have arrived at their disorder via multiple streams of temperamental vulnerability
Meehan et al. (2008)	Age 7–10 years Total, *n* = 42 (27 boys, 15 female) ODD, CD, anxiety disorder, depressive disorder	Schedule for affective disorders and schizophrenia for school aged children Rorschach inkblot method	Children with greater ADHD symptoms displayed lower scores on variables indicating internal resources for emotional self-regulation and stress tolerance relative to a comparison group
Melnick and Hinshaw (2000)	Age 6–12 years ADHD, *n* = 45; controls, *n* = 34	Video-taped family interactions and summer camp interactions rated by trained observers Parenting behaviors rated by assessors based on positive/negative parenting and parents’ ability to self-regulate their own emotions Peer-nominated sociometric assessments	Differences in emotion regulation were not attributable to severity of ADHD symptoms A generalized difficulty with emotion regulation was not shown in the ADHD population, but was a specific vulnerability among the highly aggressive subgroup Emotion regulation abilities were modestly related to underlying problems with impulse control and hyperactivity The aggressive subgroup of boys with ADHD had a slight tendency toward more intense emotional reactivity, however, emotional reactivity was predictive of neither core ADHD symptomatology nor social outcomes
Musser et al. (2011)	Age 7–9 years ADHD, *n* = 32 (ADHD-C, *n* = 19; ADHD-I, *n* = 13 [AD, 10 %; OD, 13.3 %; SD, 13.3 %]); controls, *n* = 34 (AD, 5.6 %; OD, 2.8 %; SD, 2.8 %)	Conners’ Rating Scale Strengths and Difficulties Questionnaire Wechsler’s intelligence scale for children—4th version Wechsler individual achievement test–2nd edition Cardiac pre-ejection period Respiratory sinus arrhythmia	Children with ADHD displayed a stable pattern of elevated parasympathetic activity across all task conditions compared to baseline ADHD in childhood was associated with abnormal parasympathetic mechanisms involved in emotion regulation
Musser et al. (2013)	Age 7–11 years ADHD-C, *n* = 75 ([ADHD only, *n* = 54; MD, 3.7 %; AD, 23.9 %; ODD 24.7 %; tic disorder, 3.7 %; SD, 7.1 %]; [ADHD + low prosocial, *n* = 21; MD, 3.7 %, AD, 4.7 %; ODD 23.8 %; SD, 4.7 %]); controls, *n* = 75 (MD, 2.7 %; AD, 21.3 %; ODD 8.1 %; SD, 5.4 %)	Strengths and Difficulties Questionnaire Prosocial Behavior Scale Cardiac pre-ejection period Respiratory sinus arrhythmia	ADHD-typical prosocial group displayed atypically elevated parasympathetic reactivity (emotion dysregulation) during positive induction, along with increased sympathetic activity (elevated arousal) ADHD-low prosocial group displayed reduced parasympathetic reactivity and reduced sympathetic activity (low emotional arousal) across baseline and task conditions Both ADHD groups had altered patterns of autonomic functioning In addition to clinical heterogeneity, results suggested that ADHD is heterogeneous with regard to physiological indices of emotion and regulation
Posner et al. (2013)	Age 7–12 years ADHD, *n* = 22 (ADHD-C, *n* = 19; ADHD-I, *n* = 3 [ODD, n = 6; SAD, *n* = 1]; controls, *n* = 20	DuPaul Barkley ADHD Rating Scale Conners’ Parent Rating Scale–Revised Child behavior Checklist Hollingshead index of social position Edinburgh Handedness Inventory Wechsler Abbreviated Scale of Intelligence Resting-state functional connectivity MRI	Children with ADHD had reduced connectivity in two neural circuits: one underlying executive attention and the other emotional regulation A double dissociation was seen between these two neural circuits and their behavioral correlates Reduced connectivity in the executive attention circuit correlated with executive attention deficits, but not with emotional lability Reduced connectivity in the emotional regulation circuit correlated with emotional lability, but not with executive attention deficits
Rosen et al. (2013)	*Study 1* Age 8–11 years ADHD, *n* = 11; (ADHD-C, *n* = 9; ADHD-I, *n* = 2); comorbidities, *n* = 9, including mood, anxiety and behavioral	Ecological momentary assessment Recurrence quantification analysis Emotion regulation checklist Child behavior Checklist Children’s Depression Inventory–2nd edition	A link was established between nonlinear patterning of emotional variability over time and conventional measures of emotional functioning A 4-week parent-reported, but not child-reported, ecological momentary assessment-based protocol was a feasible means of assessing emotion dysregulation in children
	*Study 2 (subset of study 1)* Age 8–11 years ADHD, *n* = 5 (ADHD-C, *n* = 4; ADHD-I, *n* = 1)	As above—ecological momentary assessment completed by the child (self-reported)	
Semrud-Clikeman et al. (2010)	Age 9–17 years ADHD, *n* = 64 (ADHD-C, *n* = 21; ADHD-I, *n* = 28; AS, *n* = 15); controls, *n* = 32	Woodcock–Johnson cognitive battery III Delis–Kaplan executive functioning system The structured interview for diagnostic assessment of children Behavior Rating Inventory of Executive Function	Children with ADHD-C had significantly more difficulty with behavioral regulation and more difficulty in emotional control compared with children with ADHD-PI or healthy controls The Behavior Rating Inventory of Executive Function indicated significant problems in the areas of flexibility, shifting, and inhibition for children with ADHD-C The ability to be flexible and to shift from one activity to the next was important for social and emotional functioning and development
Seymour et al. (2012)	Age 10–14 years ADHD, *n* = 37; controls, *n* = 32 ODD, CD, disruptive behavior disorders	Behavioral assessment system for children or adolescents-parent report Children’s Depression Inventory Emotion regulation checklist Difficulties in Emotion Regulation Scale	ADHD status was positively related to both depressive symptoms and emotion dysregulation, and depressive symptoms were positively related to emotion dysregulation Youth in the ADHD group demonstrated significantly higher levels of depression and emotion dysregulation than those in the non-ADHD control group
Seymour et al. (2014)	Age 9–12 years *n* = 227 (156 boys)	Emotion regulation checklist (maternal report) Revised Child Anxiety and Depression Scales (self-report)	Emotion regulation in youth with ADHD was monitored over a 3-year study Emotion regulation was identified as a potential mechanism linking ADHD and depressive symptoms in these individuals
Sjowall et al. (2013)	Age 7–13 years ADHD, *n* = 102 [ADHD-C, 70 %; ADHD-HI, 4 %; ADHD-PI, 26 %; (ODD or CD, 46 %; GAD/anxiety NOS, 7 %; OCD, 1 %; TS, 4 %)]; controls, *n* = 102	‘Find the phone’ Children’s size-ordering task Digit span Go/No-Go task Navon-like test Choice delay task Emotion Questionnaire	Children with ADHD differed significantly from controls, except for delay aversion and recognition of disgust Executive functioning, reaction time variability, and emotional functioning all contributed independently to distinguishing between children with ADHD and controls Emotional functioning appears to be an area of importance for ADHD that needs to be incorporated into future theoretical models
Sorensen et al. (2011)	Age 7–9 years ADHD, *n* = 23; ADHD + anxiety, *n* = 11; anxiety, *n* = 24 CD, TS, OCD, mania, depression, dysthymia excluded from study	Behavior Rating Inventory of executive function	Behavioral dysregulation in ADHD children was aggravated by comorbid anxiety
Wahlstedt et al. (2008)	Age 4–6 years Total, *n* = 87 (ADHD + EF, *n* = 16; ADHD, *n* = 19; EF, *n* = 17); controls, *n* = 35	Stroop test Go/No-Go test ‘Find the pig’ Digit span test Number of items in a stated category Parent-reported Prosocial Orientation Scale questionnaire [Rydell et al. (2003)] Strengths and Difficulties Questionnaire	Only ADHD symptoms predicted other aspects of socioemotional functioning such as dysfunctional emotional regulation and lower levels of social competence Both ADHD symptoms and impaired executive function acted as early predictors of problem behaviors
Walcott et al. (2004)	Age 6–11 years ADHD, *n* = 26; controls, *n* = 23	Stop signal reaction time Competitive puzzle task: *race to complete Lego puzzle with missing piece* Recorded and coded by trained observers Emotion control condition (half of participants told to hide emotions)	Boys with impulsive ADHD displayed greater behavioral disinhibition and were less effective at emotion regulation than comparison boys Boys with ADHD were unsuccessful in masking their emotions or changing their emotion regulation or disinhibition even when instructed to do so Comparison boys were more successful at emotion regulation when given instruction to self-regulate A small, but significant, relation was seen between executive behavior disinhibition and subsequent deficits in self-regulation of emotion
Wiersema and Roeyers (2009)	Age 8–13 years ADHD, *n* = 10 (ADHD-C, *n* = 8; ADHD-HI, *n* = 2); controls, *n* = 16	Disruptive Behavior Disorder Rating Scale Effortful Control Scale Attentional Control Scale Go/No-Go task Electroencephalogram Event-related potential	The P3 wave is an event-related potential component elicited in the process of decision making; the N2 is known as a mismatch detector, but has also been found to reflect executive cognitive control functions Children scoring high on ADHD symptoms made more errors of commission and showed smaller No-Go P3 amplitudes Effortful Control Scale and Attentional Control Scale scores were not related uniquely to inattention or hyperactive–impulsive symptoms Effortful Control was implicated in ADHD symptomatology, as children scoring high on ADHD symptoms scored low on Effortful Control questionnaires, made more errors of commission, and showed smaller No-Go P3 amplitudes

*AD* anxiety disorder, *ADHD*-*C* combined-type ADHD, *ADHD*-*HI* predominantly hyperactive–impulsive ADHD, *ADHD*-*I* inattentive ADHD, *ADHD*-*PI* predominantly inattentive ADHD, *AS* Asperger syndrome, *BD* bipolar disorder, *CD* conduct disorder, *DCD* developmental coordination disorder, *EF* executive function, *FAS* Fetal Alcohol Syndrome, *MRI* magnetic resonance imaging, *GAD* generalized anxiety disorder, *MD* mood disorder, *NOS* not otherwise stated, *OCD* obsessive compulsive disorder, *OD* oppositional disorder, *ODD* oppositional defiant disorder, *SAD* social anxiety disorder, *SD* sleep disorder, *SMD* severe mood dysregulation, *StDev* standard deviation, *TS* Tourette syndrome

## Deficits in emotional control

According to Barkley’s model (Barkley 1997a, b), individuals with ADHD exhibit deficits in inhibition when faced with emotionally charged situations, leading to greater emotional reactivity compared with those without ADHD. In support, children and adolescents (aged 6–15 years) with ADHD were significantly more emotionally reactive than control participants to positive and negative emotionally charged immediate and future challenges (Jensen and Rosen 2004). Similarly, in a study in boys aged 6–12 years challenged with a regulation and control task consisting of a competitive unsolvable Lego puzzle task in which half of the participants were told to hide their feelings, individuals with ADHD were found to have greater behavioral disinhibition and were less able to regulate their emotions, even when prompted to do so, than healthy controls (Walcott and Landau 2004).

Deficit in emotional self-regulation was assessed in a large cohort of 216 children with ADHD and 142 siblings using Conners’ Rating Scale (Parent version) (Anastopoulos et al. 2011). A greater proportion of children with ADHD (46.92 %) displayed high levels of emotional lability than controls (15.38 %). Children with ADHD also showed significantly inferior outcomes in functional impairment and comorbidities, especially aggression and depression, compared with controls. Emotional lability in ADHD was associated with worse outcomes and with multiple-treatment utilization (e.g., use of medication, parent training, and individual therapy). The self-regulation of affect was compared in boys with ADHD and unaffected peers (Braaten and Rosen 2000). Compared with controls, boys with ADHD were found to be less empathic (based on the empathy response task) and had higher scores on the negative emotions subscale (scores for sadness, anger, and guilt) of the Emotional Intensity Scale for Children, although the level of emotional intensity reported by the children did not differ between controls and those with ADHD.

Evidence for a relationship between disturbed emotional self-regulation (the capacity to regulate affect and sustain attention) and the severity of symptoms of ADHD was provided using the Rorschach inkblot method (Meehan et al. 2008). Although without a formal diagnosis of ADHD, children with higher ADHD symptom scores exhibited reduced access to internal resources compared with the comparison group. Furthermore, children with more severe ADHD symptoms generated fewer human movement responses than those with fewer symptoms, suggesting that the former group may have shied away from social exchange. These results appeared to point to a deficit in emotional self-regulation in children with greater symptoms of ADHD. In an investigation of the impact of the subtype of ADHD on emotional self-regulation, children with the combined subtype of ADHD displayed emotional dysregulation (high intensity and high levels of positive and negative behavior) and aggressive behavior, whereas those with the inattentive subtype of ADHD displayed impaired social functioning (social passivity and deficits in social knowledge), but did not exhibit problems with emotional regulation (Maedgen and Carlson 2000). In contrast, a study that evaluated emotional regulation using family interactions during a frustration-inducing task found that poor emotion regulation was not linked to core ADHD symptomatology, but was associated with an aggressive subgroup of boys with ADHD (Melnick and Hinshaw 2000).

Although rating scales are important tools in understanding emotional lability in individuals with ADHD, most are not designed to examine how emotional dysregulation varies over time as children with the disorder attempt to maintain emotional homeostasis during their daily lives. The ecological momentary assessment (EMA) utilizes a handheld device both to prompt parents to enter assessments of their child’s emotional state and to receive them and, therefore, has the potential to assess both discrete and temporal facets of emotional dysregulation. In a small (*n* = 11), exploratory study in children (aged 8–11 years) with ADHD, parents reported data about their child’s emotional status three times daily (before school, after school, and in the evening) for 28 days (a total of 84 assessments) with outcomes correlated with those of the emotion regulation checklist, the child behavior checklist, and the Children’s Depression Inventory 2nd Edition supporting the validity and utility of the parent-reported EMA (Rosen et al. 2013). Results from a small (*n* = 5) parallel study did not support the validity of child-reported EMA, perhaps because self-report was problematic in emotionally distressed children (Rosen et al. 2013).

## Impairments in executive functioning

Poor executive functioning, which includes the maintenance of future goals, inhibitory control, working memory, and planning, has been linked to the impairment associated with ADHD (Barkley 2001; Willcutt et al. 2005). Accordingly, in 21 boys aged 7–10 years diagnosed with combined or predominantly hyperactive–impulsive subtypes of ADHD and 43 age-matched controls, several measures of executive function, including tests of inhibition, working memory, internalization of speech, self-regulation of emotional arousal, and reconstitution, but not the repetition of the hand movements test relating to working memory, were all found to be independent predictors of ADHD (Berlin et al. 2004).

A possible link between the symptoms and impaired executive function in individuals with ADHD and several socioemotional behaviors, including emotional dysregulation, was investigated in a longitudinal, 2-year study in 87 children (aged 4–6 years) with ADHD and/or impaired executive function (Wahlstedt et al. 2008). Executive function was assessed using measures of inhibition, spatial and verbal working memory, verbal fluency, and intelligence; behavioral assessments included symptoms, emotional regulation, social competence, and internalizing problems. Although both symptoms and impaired executive function were evident for the entire study period, only symptoms at study initiation predicted emotional dysregulation and lower levels of social competence at study end, suggesting that symptoms and impaired executive function are not entirely co-dependent facets of the disorder.

The Behavior Rating Inventory of Executive Function (BRIEF) is a generic instrument used to assess executive function in children and adolescents with ADHD (McCandless and O’Laughlin 2007; Toplak et al. 2009). The BRIEF comprises two indices, Behavioral Regulation and Metacognition, with the former incorporating elements of emotional dysregulation, including inhibition (the ability to resist impulses and stop one’s behavior at the appropriate time), shifting (the ability to make transitions, tolerate change, problem-solve flexibly, and switch one’s attention from one topic to another), and emotional control (the influence of executive functions on the expression and regulation of emotions) (Isquith and Gioia 2008). Sorensen et al. (2011) compared parent-reported BRIEF outcomes in children with ADHD (*n* = 23), anxiety disorder (*n* = 24), ADHD with comorbid anxiety disorder (*n* = 11), and a healthy control group. ADHD was associated with impairments on the inhibit, emotional control, and working memory elements of the BRIEF (Sorensen et al. 2011). Semrud-Clikeman et al. (2010) also used the BRIEF, alongside neuropsychological measures, to evaluate executive function in children with predominantly inattentive ADHD (*n* = 28), combined-type ADHD (*n* = 21), Asperger syndrome (*n* = 15), and controls (*n* = 32) (Semrud-Clikeman et al. 2010). Overall, this study found that the clinical groups exhibited more difficulty in measures of executive function than controls, although the pattern of impairment across the three clinical groups varied across individual neuropsychological measures and individual BRIEF scales. For example, in the behavioral regulation scales of the BRIEF, scores for children with the combined subtype of ADHD were significantly higher than controls for emotional control, inhibit, and shift, whereas scores for children with the predominantly inattentive subtype of ADHD significantly differed from controls in the inhibit scale only. In contrast, children with combined and predominantly inattentive subtypes of ADHD exhibited significantly greater difficulty than controls in all of the metacognition scales of the BRIEF. Differences in the executive functioning in the clinical groups, including ADHD subtypes, have implications for therapeutic interventions. Furthermore, the observation that the severity of the symptoms of ADHD (or Asperger syndrome) was significantly related to difficulties with behavioral regulation and metacognition suggests that symptom-related behaviors interact with executive processes.

## Emotional components of comorbidities

Evidence that emotional dysregulation may be an important component of ADHD, particularly as it presents in adolescence, was provided by a longitudinal study in which individuals diagnosed with ADHD with or without comorbid disruptive behavior disorders during childhood (aged 7–11 years) were followed up 10 years later (Harty et al. 2009). High levels of aggression associated with increased emotionality in the form of anger were reported in adolescents diagnosed with ADHD during childhood, with most of the group differences at follow-up in verbal aggression and anger (but not physical aggression) accounted for by the persistence of ADHD symptoms into adolescence.

A positive correlation between ADHD and both depressive symptoms and emotional dysregulation in a small study of youths aged 10–14 years with (*n* = 37) or without (*n* = 32) ADHD led to the hypothesis that emotional dysregulation may link ADHD in childhood with the development of depression in later life (Seymour et al. 2012). Evidence of a link between ADHD symptoms, emotion regulation ability, and depressive symptoms was subsequently confirmed in a larger cohort of 227 youths, of whom 27 met DSM-IV diagnostic criteria for ADHD and were monitored over a 3-year period (Seymour et al. 2014). Structural equation modeling suggested that emotional dysregulation was a significant mechanism underlying the relationship between ADHD and the development of later depression. Although both hyperactive–impulsive symptoms and inattentive symptoms predicted the development of depression, the symptoms of hyperactivity–impulsivity were the more robust predictors of emotional dysregulation. Differences in the pathophysiology of emotional dysregulation in ADHD and other psychiatric disorders were suggested by a functional imaging study in which children with ADHD, unlike those with bipolar disorder or severe mood disorder, displayed hyperactivity of the left amygdala when rating subjective fear of neutral faces suggesting different neural correlates of face-emotion processing in patients with ADHD compared with the other disorders (Brotman et al. 2010).

## Neurophysiology and imaging correlates of emotional dysregulation

The amygdala is implicated in the regulation of emotion and the relationship between intrinsic functional connectivity (iFC) of amygdala circuits, and emotion regulation deficit in children aged 6–13 years with ADHD (*n* = 63) and healthy controls (*n* = 19) was evaluated using functional magnetic resonance imaging (fMRI) (Hulvershorn et al. 2014). Within the ADHD group, higher levels of emotional lability, determined using the Emotional Lability subscale of the Conners’ Parent Rating Scale–Revised, long version, were associated with greater positive iFC between the amygdala and the anterior cingulate cortex, and with negative iFC between the amygdala and the posterior insula/superior temporal gyrus. Differences between the ADHD subgroup with low levels of emotional lability and healthy controls were small and nonsignificant, suggesting that the abnormal functional connectivity was specific to emotional lability rather than to other ADHD symptoms.

Effortful control, or the self-regulation aspects of temperament, was investigated in children with ADHD (*n* = 10) and typically developing controls (*n* = 16) using a combination of psychosocial (the Effortful Control Scale and the Attentional Control Scale) and physiological [electroencephalographic event-related potentials (ERPs)] measures during the administration of the Go/No-Go task (Wiersema and Roeyers 2009). ADHD symptoms were associated with low scores on the rating scales, errors of commission in the Go/No-Go task, and small No-Go P3 amplitudes, suggesting that effortful control is implicated in ADHD. ERP evidence of atypical emotional processing was also observed during a facial emotional processing task in adolescent boys with ADHD (*n* = 31) compared with controls without ADHD (*n* = 32) (Gow et al. 2013). Negative facial expressions were associated with an exaggerated P2 wave in those with ADHD; N2 and N4 waves were also atypical in this group. Furthermore, ERP abnormalities in emotional processing were significantly associated with lower plasma omega-3 fatty acids levels in the ADHD group, suggesting that lower plasma long-chain polyunsaturated fatty acids are associated with impaired emotional processing in ADHD.

## Autonomic correlates of emotional dysregulation

Respiratory sinus arrhythmia (RSA) is the natural variation in heart rate that occurs during breathing, whereby heart rate increases during inhalation and decreases during exhalation and is an indirect biomarker of vagal tone. RSA is particularly pronounced in children, and abnormal RSA has been linked to emotional dysregulation in childhood psychopathologies (Beauchaine et al. 2013). In a study of children with ADHD (*n* = 32) and typically developing controls (*n* = 34), physiological recordings were conducted during a task that required participants to view an emotion-laden film while mimicking (induction) or masking (suppression) the emotion of the main character (Musser et al. 2011). Healthy controls exhibited systematic variation in RSA, which depended on emotional valence (more activation for negative emotion, reduced activation for positive emotion) and task demand (more activation for suppression than induction), whereas children with ADHD displayed a stable pattern of elevated parasympathetic activity across all task conditions compared to baseline. No group differences in cardiac pre-ejection period (PEP, an indicator of sympathetic activity) were observed. Added complexity was provided by a subsequent study using similar methodology in which participants were classified into three groups: children with ADHD and low prosocial behavior such as callousness or unemotional traits (*n* = 21), those with ADHD with developmentally appropriate prosocial behavior (*n* = 54), and typically developing children (*n* = 75) (Musser et al. 2013). In the ADHD group with developmentally appropriate prosocial behavior, positive induction was associated with atypically elevated parasympathetic (RSA) reactivity during positive induction, along with increased sympathetic (PEP) activity across conditions. In contrast, children with ADHD and low prosocial behavior displayed both reduced parasympathetic reactivity and reduced sympathetic activity across baseline and task conditions. Thus, both ADHD groups exhibited altered patterns of autonomic functioning, but in two distinct forms.

The possible utility of this form of physiological testing in identifying children at risk of emotional dysregulation was investigated in a cohort of 99 young children (aged 4–6 years) with ADHD during three conditions (baseline, reward task, and block building) (Beauchaine et al. 2013). Children with low RSA at baseline and greater RSA withdrawal during block building exhibited poorer regulation of emotion and scored lower for prosocial behavior than their peers. Furthermore, lengthened cardiac PEP at rest and reduced PEP reactivity to incentives were associated with increased levels of conduct problems and aggression.

## Multiple pathway models of ADHD

The heterogeneity of ADHD is reflected in the proposal of various multiple pathway models for the disorder. One example proposes that executive deficits lead to symptoms of inattention and that reward–response deficits lead to hyperactivity (Sonuga-Barke 2002, 2005). A second example proposes that effortful control is associated with the core symptoms of ADHD, negative emotionality with comorbid antisocial or disruptive behavior (Nigg et al. 2004). This suggests that the neuropsychological deficits that underpin the symptoms of ADHD may be different from those leading to disruptive behavior problems was investigated by examining the personality–temperament traits of reactive and effortful control, resiliency, and emotionality in 179 children aged 6–12 years with ADHD (*n* = 92), without ADHD (*n* = 52), or with borderline or not otherwise specified ADHD (*n* = 35) (Martel and Nigg 2006). The results support a model in which regulation problems contribute to the emergence of symptoms of inattention–disorganization and reactive or motivational control problems contribute to the emergence of hyperactivity–impulsivity symptoms, whereas negative affect was associated with oppositional defiance. Thus, children exhibiting regulation deficits and reactive motivational behaviors may be at particular risk of the development of ADHD.

The contribution of deficits in different neuropsychological domains to ADHD was examined in children (aged 7–12 years) with ADHD (*n* = 102) and controls (individually matched for age and sex) using assessments of executive function (working memory, inhibition, and shifting), delay aversion, reaction time variability, and emotion regulation and recognition (Sjowall et al. 2013). Children with ADHD differed significantly from controls on all measures, except for delay aversion and recognition of disgust. Executive functioning, reaction time variability, and emotional functioning all contributed independently to distinguishing between children with ADHD and controls. This study, therefore, supports the concept of ADHD as a heterogeneous disorder and highlights the importance of emotional functioning to conceptual models of the disorder.

Physiological evidence for a dissociation between attentional and affective neural pathways in individuals with ADHD was generated using resting-state functional connectivity MRI in medication-naïve children with (*n* = 22) or without (*n* = 20) ADHD. Neural connectivity in neural circuits underlying executive function and emotional regulation was found to be reduced in participants with the disorder compared with controls (Posner et al. 2013). Interestingly, reduced connectivity in the executive attention circuit was related to executive attention deficits, but not to emotional lability, whereas reduced connectivity in the emotional regulation circuit related to emotional lability but not to executive attention deficits. Thus, these imaging data add further weight to the suggestion that multiple pathways underpin the pathophysiology of ADHD.

## Conclusions

Understanding of emotional dysregulation in children with ADHD is not complete, and there is currently no single definition for the phenomenon. Neuropsychological tests of emotional control (impulsivity, self-regulation of positive and negative emotions, and executive function) indicate that the processing of emotional stimuli is impaired in ADHD. Most descriptions of emotional dysregulation in ADHD include the concepts of an inability to modulate emotional responses and excessive reactions to a particular emotional trigger that would be considered inappropriate for the developmental age of the individual and the social setting. Inappropriate responses may be both internalized (e.g., the individual may be withdrawn, moody, or sad) or externalized (e.g., they may be emotionally volatile, aggressive, argumentative, and even physical). Furthermore, evidence continues to emerge that deficits in more than one neurological pathway are responsible for emotional dysregulation in ADHD.

**Fig. 2:**
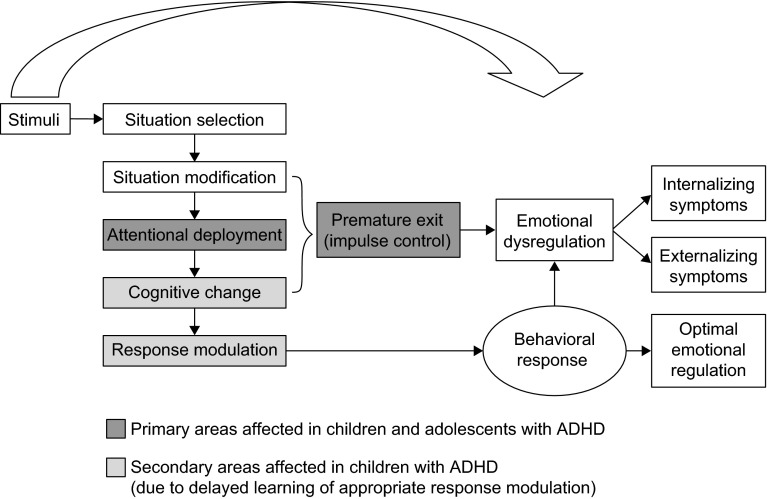
Conceptual model for emotional dysregulation in children with ADHD. *ADHD* attention-deficit/hyperactivity disorder. Developed and adapted from Gross (1998)

Figure 2, adapted from Gross (1998), presents a possible conceptual model which attempts to visualize how extrinsic or intrinsic stimuli may lead to emotional dysregulation, resulting in internalizing or externalizing symptoms in children with ADHD. Early-life emotion regulation is managed largely by extrinsic *situation selection* (e.g., parents organizing their child’s daily routine), but as children grow older, they develop their own regulatory processes. *Situation modification* is an attempt, either extrinsically or intrinsically, to alter the subsequent course of the emotional response. *Attention deployment* is where the individual selects the aspect of the situation to focus on in order to help control the emotional response. *Cognitive change* is where the individual changes their perception of the situation (intrinsic), or they alter someone else’s perception, or have their own perception changed by someone else (extrinsic). *Response modulation* refers to the attempts to directly influence the response tendencies through the action of drugs, food, exercise and relaxation, etc. or by the modulation of emotion-expressive behavior. This process may not be learned at age appropriate times, and individuals with ADHD may have poor attention deployment and impulse control, thereby leading to premature exit from the cognitive process and resulting in emotional dysregulation.

Although caregivers and patients with ADHD constantly search for explanations for emotional lability such as that described in the two case examples at the beginning of this article, there is currently no gold standard for assessing emotional dysregulation in ADHD. In many cases, individuals with ADHD and significant impairment from emotional dysregulation do not meet DSM-5 criteria for a distinct co-morbid diagnosis. Having clinically useful measures of emotional dysregulation, and furnishing clinicians with a robust definition, could help caregivers and patients to improve their understanding of the condition. Defining and measuring emotional dysregulation on a clinical level should be a priority. Novel or new applications of neuropsychological instruments, neurophysiological and imaging techniques, and biomarkers of emotional dysregulation in children with ADHD are emerging, and however, more research is needed to develop reliable and universally accepted measures in affected children and to determine which deficits are particularly problematic at a functional level in terms of their daily life and social, emotional, and cognitive development

Once such measures are developed, they could be used to test the efficacy of potential treatments in emotional dysregulation. Clinically, parents report that there is improvement in emotional regulation when ADHD is treated pharmacologically, perhaps through improvements in the areas of impulse control and attentional deployment. ADHD pharmacotherapy does not, however, address the delayed development of cognitive control skills in individuals with ADHD. Hence, in addition to pharmacotherapy, treatment should focus on the learning of cognitive skills throughout the developmental process.

## References

[CR1] American Psychiatric Association (2013) Diagnostic and statistical manual of mental disorders, fifth edition (DSM-5). American Psychiatric Publications

[CR2] Anastopoulos AD, Smith TF, Garrett ME, Morrissey-Kane E, Schatz NK, Sommer JL, Kollins SH, Ashley-Koch A (2011). Self-regulation of emotion, functional impairment, and comorbidity among children with AD/HD. J Atten Disord.

[CR3] Barkley RA (1997). Attention-deficit/hyperactivity disorder, self-regulation, and time: toward a more comprehensive theory. J Dev Behav Pediatr.

[CR4] Barkley RA (1997). Behavioral inhibition, sustained attention, and executive functions: constructing a unifying theory of ADHD. Psychol Bull.

[CR5] Barkley RA (2001). The executive functions and self-regulation: an evolutionary neuropsychological perspective. Neuropsychol Rev.

[CR6] Barkley RA (2010). Deficient emotional self-regulation: a core component of attention-deficit/hyperactivity disorder. J ADHD Relat Disord.

[CR7] Beauchaine TP, Gatzke-Kopp L, Neuhaus E, Chipman J, Reid MJ, Webster-Stratton C (2013). Sympathetic- and parasympathetic-linked cardiac function and prediction of externalizing behavior, emotion regulation, and prosocial behavior among preschoolers treated for ADHD. J Consult Clin Psychol.

[CR8] Berlin L, Bohlin G, Nyberg L, Janols L-O (2004). How well do measures of inhibition and other executive functions discriminate between children with ADHD and controls?. Child Neuropsychol.

[CR9] Braaten EB, Rosen LA (2000). Self-regulation of affect in attention deficit-hyperactivity disorder (ADHD) and non-ADHD boys: differences in empathic responding. J Consult Clin Psychol.

[CR10] Brotman MA, Rich BA, Guyer AE, Lunsford JR, Horsey SE, Reising MM, Thomas LA, Fromm SJ, Towbin K, Pine DS, Leibenluft E (2010). Amygdala activation during emotion processing of neutral faces in children with severe mood dysregulation versus ADHD or bipolar disorder. Am J Psychiatry.

[CR103] Gordon M (1995) How to operate an ADHD clinic or subspeciality practice. DeWitt, New York

[CR11] Gow RV, Sumich A, Vallee-Tourangeau F, Crawford MA, Ghebremeskel K, Bueno AA, Hibbeln JR, Taylor E, Wilson DA, Rubia K (2013). Omega-3 fatty acids are related to abnormal emotion processing in adolescent boys with attention deficit hyperactivity disorder. Prostaglandins Leukot Essent Fatty Acids.

[CR12] Gross JJ (1998). The emerging field of emotion regulation: an integrative review. Rev Gen Psychol.

[CR13] Harty SC, Miller CJ, Newcorn JH, Halperin JM (2009). Adolescents with childhood ADHD and comorbid disruptive behavior disorders: aggression, anger, and hostility. Child Psychiatry Hum Dev.

[CR14] Hulvershorn LA, Mennes M, Castellanos FX, Di Martino A, Milham MP, Hummer TA, Roy AK (2014). Abnormal amygdala functional connectivity associated with emotional lability in children with attention-deficit/hyperactivity disorder. J Am Acad Child Adolesc Psychiatry.

[CR15] Isquith PK, Gioia GA (2008) Behavior Rating Inventory of Executive Function^®^ Feedback Report. http://www.psychassessments.com.au/products/280/prod280_report1.pdf Accessed: 22 Sept 2015

[CR16] Jensen SA, Rosen LA (2004). Emotional reactivity in children with attention-deficit/hyperactivity disorder. J Atten Disord.

[CR17] Karalunas SL, Fair D, Musser ED, Aykes K, Iyer SP, Nigg JT (2014). Subtyping attention-deficit/hyperactivity disorder using temperament dimensions: toward biologically based nosologic criteria. JAMA Psychiatry.

[CR18] Laufer M, Denhoff E, Solomons G (1957). Hyperkinetic impulse disorder in children’s behavior problems. Psychosom Med.

[CR19] Maedgen JW, Carlson CL (2000). Social functioning and emotional regulation in the attention deficit hyperactivity disorder subtypes. J Clin Child Psychol.

[CR20] Martel MM, Nigg JT (2006). Child ADHD and personality/temperament traits of reactive and effortful control, resiliency, and emotionality. J Child Psychol Psychiatry.

[CR21] McCandless S, O’Laughlin L (2007). The Clinical Utility of the Behavior Rating Inventory of Executive Function (BRIEF) in the diagnosis of ADHD. J Atten Disord.

[CR22] Meehan KB, Ueng-McHale JY, Reynoso JS, Harris BH, Wolfson VM, Gomes H, Tuber SB (2008). Self-regulation and internal resources in school-aged children with ADHD symptomatology: an investigation using the Rorschach inkblot method. Bull Menn Clin.

[CR23] Melnick SM, Hinshaw SP (2000). Emotion regulation and parenting in AD/HD and comparison boys: linkages with social behaviors and peer preference. J Abnorm Child Psychol.

[CR24] Musser ED, Backs RW, Schmitt CF, Ablow JC, Measelle JR, Nigg JT (2011). Emotion regulation via the autonomic nervous system in children with attention-deficit/hyperactivity disorder (ADHD). J Abnorm Child Psychol.

[CR25] Musser ED, Galloway-Long HS, Frick PJ, Nigg JT (2013). Emotion regulation and heterogeneity in attention-deficit/hyperactivity disorder. J Am Acad Child Adolesc Psychiatry.

[CR26] Nigg JT, Goldsmith HH, Sachek J (2004). Temperament and attention deficit hyperactivity disorder: the development of a multiple pathway model. J Clin Child Adolesc Psychol.

[CR27] Posner J, Rauh V, Gruber A, Gat I, Wang Z, Peterson BS (2013). Dissociable attentional and affective circuits in medication-naive children with attention-deficit/hyperactivity disorder. Psychiatry Res.

[CR28] Rhodes SM, Coghill DR, Matthews K (2005). Neuropsychological functioning in stimulant-naive boys with hyperkinetic disorder. Psychol Med.

[CR101] Ricard M, Kamberk-Kilicci M (1995). Children's empathic responses to emotional complexity. Int J Behav Dev.

[CR29] Rosen PJ, Epstein JN, Van Orden G (2013). I know it when I quantify it: ecological momentary assessment and recurrence quantification analysis of emotion dysregulation in children with ADHD. Atten Defic Hyperact Disord.

[CR100] Rydell AM, Berlin L, Van Bohlin G (2003). Emotionality, emotion regulation, and adaptation among 5- to 8-year-old children. Emot.

[CR30] Semrud-Clikeman M, Walkowiak J, Wilkinson A, Butcher B (2010). Executive functioning in children with Asperger syndrome, ADHD-combined type, ADHD-predominately inattentive type, and controls. J Autism Dev Disord.

[CR31] Seymour KE, Chronis-Tuscano A, Halldorsdottir T, Stupica B, Owens K, Sacks T (2012). Emotion regulation mediates the relationship between ADHD and depressive symptoms in youth. J Abnorm Child Psychol.

[CR32] Seymour KE, Chronis-Tuscano A, Iwamoto DK, Kurdziel G, Macpherson L (2014). Emotion regulation mediates the association between ADHD and depressive symptoms in a community sample of youth. J Abnorm Child Psychol.

[CR33] Shaw P, Stringaris A, Nigg J, Leibenluft E (2014). Emotion dysregulation in attention deficit hyperactivity disorder. Am J Psychiatry.

[CR34] Sjowall D, Roth L, Lindqvist S, Thorell LB (2013). Multiple deficits in ADHD: executive dysfunction, delay aversion, reaction time variability, and emotional deficits. J Child Psychol Psychiatry.

[CR35] Sonuga-Barke EJS (2002). Psychological heterogeneity in AD/HD—a dual pathway model of behaviour and cognition. Behav Brain Res.

[CR36] Sonuga-Barke EJ (2005). Causal models of attention-deficit/hyperactivity disorder: from common simple deficits to multiple developmental pathways. Biol Psychiatry.

[CR37] Sorensen L, Plessen KJ, Nicholas J, Lundervold AJ (2011). Is behavioral regulation in children with ADHD aggravated by comorbid anxiety disorder?. J Atten Disord.

[CR38] Stringaris A (2011). Irritability in children and adolescents: a challenge for DSM-5. Eur Child Adolesc Psychiatry.

[CR39] Stringaris A, Goodman R (2009). Three dimensions of oppositionality in youth. J Child Psychol Psychiatry.

[CR40] Toplak ME, Bucciarelli SM, Jain U, Tannock R (2009). Executive functions: performance-based measures and the behavior rating inventory of executive function (BRIEF) in adolescents with attention deficit/hyperactivity disorder (ADHD). Child Neuropsychol.

[CR41] Wahlstedt C, Thorell LB, Bohlin G (2008). ADHD symptoms and executive function impairment: early predictors of later behavioral problems. Dev Neuropsychol.

[CR42] Walcott CM, Landau S (2004). The relation between disinhibition and emotion regulation in boys with attention deficit hyperactivity disorder. J Clin Child Adolesc Psychol.

[CR43] Ward MF, Wender PH, Reimherr FW (1993). The Wender Utah Rating Scale: an aid in the retrospective. Am J Psychiatry.

[CR44] Wiersema JR, Roeyers H (2009). ERP correlates of effortful control in children with varying levels of ADHD symptoms. J Abnorm Child Psychol.

[CR45] Willcutt EG, Doyle AE, Nigg JT, Faraone SV, Pennington BF (2005). Validity of the executive function theory of attention-deficit/hyperactivity disorder: a meta-analytic review. Biol Psychiatry.

